# New Journals

**Published:** 1884-05

**Authors:** 


### NEW JOURNALS.

We take great pleasure in welcoming to our exchange table the St. Louis *Periscope*, edited by Prof. E. C. Franklin, M. D., the well-known surgeon, and *The Regular Physician*, edited by Dr. A. P. Hollett, of Havana, New York.

We hope these new candidates for professional favor may meet with all the success they deserve. We would remind them that there is always *room at the top*. Let Excelsior! be their motto.

In March the *Periscope* and the *Clinical Review* were consolidated, and are henceforth to appear under the title of the St. Louis *Periscope and Clinical Review*, edited by Drs. Franklin and Valentine. Subscription, $2.00 per annum.

The *Regular Physician* will be issued quarterly; subscription, one dollar per annum.

